# Cisgenics and intragenics: boon or bane for crop improvement

**DOI:** 10.3389/fpls.2023.1275145

**Published:** 2023-11-28

**Authors:** S. N. Vasudevan, S. K. Pooja, Thota Joseph Raju, C. S. Damini

**Affiliations:** ^1^Department of Seed Science and Technology, College of Agriculture, Hassan, University of Agricultural Sciences, Bangalore, India; ^2^Department of Agricultural Biotechnology, College of Agriculture, Hassan, University of Agricultural Sciences, Bangalore, India; ^3^Chronobiology Lab, Department of Studies in Zoology, University of Mysore, Karnataka, India

**Keywords:** transgenics, transformation, selectable marker, agrobacterium-mediated, gene expression

## Abstract

Recent advances in molecular biology and genetic engineering have made it possible to increase agricultural yields when compared to conventional methods. However, lots of challenges are to be addressed due to changing climatic conditions. Although genetically modified organisms (GMOs) have proven their potential in a few crops, but needs to be explored in majority of the field/vegetable crops to overcome food and nutritional security in view of alarming population explosion. In spite of advantages from GMO crops due to the presence of foreign DNA, queries regarding their safety, environmental dangers and health effects needs to be addressed. One of the major environmental issues concerning transgenic crops is the mixing of genetic components across species that cannot hybridize naturally. Due to these limitations, new revolutionary technologies have been developed, such as intragenesis and cisgenesis for the transformation and development of superior plants. While cisgenesis entails genetic modification employing a complete copy of natural genes with their native regulatory components that only belong to sexually compatible species, intragenesis refers to the transfer of unique combinations of genes and regulatory sequence inside the same species. In cisgenesis, the donor genes are the same genes employed in conventional breeding. The two benefits of cisgenics are avoiding linkage drag and making greater use of existing gene alleles. This method significantly shortens the time it takes to breed plants by combining conventional methods with cutting-edge biotechnological tools. Because of this, plant genomes can be altered without causing drastic changes to the whole plant population and the environmental effects of cisgenic plants cannot be compared to those of transgenics. Transgenesis and cisgenesis share the same transformation methods; hence, cisgenic, intragenic and transgenic plants produced through random insertion do not pose any distinct risks with regard to host genome modifications. In contrast, using new genome techniques lessens the dangers related to potential unintentional changes to the host DNA. The use of cisgenesis and intragenesis as alternatives to transgenesis has been restricted to a small number of species due to incomplete understanding of the required regulatory sequences.

## Introduction

In traditional breeding, suitable plants are chosen and crossed to create novel types. Breeders have employed this technique to enhance crop productivity long back. Conventional breeding is time-consuming and difficult to produce new varieties in a short span of time. However, it has created crops that are suited to the area and have major benefits like higher yield, resistance to various biotic and abiotic stresses and other superior qualitative traits. Modern molecular genetics and conventional breeding are coupled in a process called marker-assisted breeding (MAB). DNA markers associated with particular phenotypic traits can be effectively used to select plants with desirable traits. Marker-assisted breeding helps in identifying desirable plants in the early generations without field tests during breeding process; it also reduces the number of plants that need to be screened. Hence, conventional breeding coupled with MAB can enhance the breeding cycle with efficient selection. MAS breed plants are inexpensive and precise than conventional breeding. However, it necessitates specific equipment, expertise and risks of changing plant traits and characteristics. Conventional breeding coupled with marker-assisted breeding can precisely contribute to food security and agricultural sustainability by creating new crop varieties with desirable features. Plants with desired traits can be crossed to create superior offspring. Genetic crossover, mutation selection and transgenesis are the main techniques used in plant breeding to create genetic variation. In conventional plant breeding, beneficial alleles from crossable species are introduced through genetic crossing; however, this approach is constrained by linkage drag and prone to fertilization barriers, resulting in a low likelihood of producing a target phenotype. Breeding is the long-term domestication process that all crop plants go through. Genetic diversity and selection are the major sources of a wide range of variation. It is anticipated that more the parental lines diverge, the more genetic influences on the characters in the segregating population. The contribution of wild species is of considerable relevance at this point because genetic heterogeneity within the gene pool of the relevant crops is in danger due to rigorous breeding and pedigree selection. The lacuna of MAB is overcome by transgenics (genes from other species), where only the gene of insert is inserted. Upon commercial release of more transgenic crops, environmental risks, ethical concerns and other potential risks to human health have risen to become major concerns around the world. As an alternative to transgenic, two new notions known as cisgenic and intragenic have emerged. Each of these methods employs genes from a gene pool that is sexually compatible, despite the fact that they are very different at the molecular level. Cisgenesis, which is the transfer of gene(s) from the host species or a closely related (sexually compatible) species along with its native regulatory sequence, potential approach of selection that keeps in mind the boundaries between species. Intragenesis, transfer of a sexually compatible gene pool, can be organised in a sense or antisense orientation. The intragenesis concept is less limited than cisgenics as genetic element rearrangement is permitted, but both of these approaches improve allele utilisation and accelerate selection without linkage drag, which requires both time and resources to complete. This makes the widespread use of cisgenics and intragenics in agriculture, as an innovative plant breeding techniques, beneficial for overcoming the difficulties faced by plants in the ambiguous climate.

Transgenesis is the process of introducing foreign DNA into the genome of an organism. It is achieved through a variety of techniques, like gene editing tools (CRISPR/Cas9) or traditional methods (genetic engineering). In agriculture, transgenesis has been widely used, especially in the creation of genetically modified (GM) crops. GM crops have been developed to possess desirable traits such as herbicide tolerance, insect resistance, drought tolerance, and improved nutritional content. These traits can be beneficial in terms of increasing yields and decreasing the need for chemical pesticides and herbicides. In 2022, there was 191.7 million hectares (472.8 million acres) of genetically modified (GM) crops worldwide, up from 67.7 million hectares (67.7%) in 2003, as per the International Service for the Acquisition of Agri-biotech Applications (ISAAA). In the past 20 years, the discussion about the purported potential health and environmental dangers has persisted unabatedly despite the rise of transgenic (GM) crops. United States (71.5 million hectares), Brazil (51.3 million hectares), Argentina (24.6 million hectares), India (11.6 million hectares), Canada (11.5 million hectares), and other nations that cultivated significant areas of GM crops in Paraguay, Pakistan, China, South Africa, Uruguay, Bolivia, and the Philippines were the top five countries in terms of acreage of GM crops in 2022. There are substantial disparities in the types of GM crops grown and the regulatory frameworks that oversee their usage, and it is vital to highlight that acceptance of GM crops varies greatly among various countries and areas. The legal status of genetically modified (GM) crops varies by location and is prohibited in certain countries. By cultivating genetically modified (GM) crops such as soybeans, maize, cotton, canola, sugar beets, alfalfa, papaya and squash, the United States has out produced all other nations since 1996. Only maize (MON 810) is permitted for cultivation in the European Union. Cotton and papaya are two of the most widely grown GM crops in China. Brazil, by cultivating GM crops such as soybeans, maize, cotton and sugarcane, stands in third place. India is the fourth-largest producer of GM crops, with cotton being the only commercial crop farmed there. The advantages and disadvantages of producing GM crops are still contested, so it’s critical to keep that in context. Conventional breeding can result in the generation of crops with desirable traits, but this process can take several years. Transgenic breeding, on the other hand, shortens this cycle because it requires less time to produce a new variety. There are a number of problems with transgenesis, with a majorly negative impact on ecological, social and health consequences that often arise unintentionally. There is a risk in the incidence of resistance to pests and weeds. Unintentional safety issues associated with GM crops include allergenicity, toxicity and changes in nutritional composition. Concerns about private dominance in agriculture have been stoked by the fact that just a handful of companies have access to transgenic technology. This may be especially harmful to small-scale farmers as it reduces the diversification of crops. It’s crucial to weigh the benefits and drawbacks of each technology and determine how to utilise it sustainably and safely because they could increase yields and reduce the need for pesticides and herbicides, but they also have undetermined adverse effects.

Transgenics regulations could end up being challenging in the future. Commercially speaking, because they were created for industrial-scale farms, the GM crops that are currently accessible may not be advantageous to small-scale or subsistence farmers in developing nations. Ecologists and conservation biologists have emphasised the necessity for thorough research on the environmental advantages and dangers of transgenic crops and discouraged their release in situations where the available scientific data on those concerns is manifestly insufficient. In order to overcome these issues while still maintaining an effective and environmentally friendly plant production, the two transformation concepts (cisgenesis and intragenesis) were developed as substitutes for transgenics. The two theories rely on genetic material from members of the same gene pool or genes from sexually compatible gene pools that have the ability to hybridise sexually. In contrast, transgenesis allows the transfer of genes and DNA sequences between different species. As a result, the gene pool used in cisgenesis and intragenesis is the same one made available for traditional breeding. Additionally, no foreign genes, including vector-backbone genes and selection marker genes, should be present in the original intragenic or cisgenic transformants or their progeny.

Dutch Christian political parties invented cisgenesis in 1999 and discovered that no gene flow from cisgenic crops to wild relatives due to cisgene isolation, which preserved species identity. In 2000, a Dutch book used “cisgenese” term developed by European scientists to develop rot-resistant cisgenic strawberries and found that growers and consumers prefer cisgenic strawberries over transgenic ones. For Cisgenic crop, GMO exemptions were passed in 2009 and 2012. Different scientists provided different definitions of cisgenesis and intragenesis (**Table 1**). The primary tenet of the early conception of cisgenesis was that the gene elements should come from the species itself, with no requirements for the coding sequence to contain introns or for the regulatory sequences to come from the same gene as the coding sequences (**Table 1**). When the current definition of the cisgenesis idea was published in international journals in 2006 (Schouten et al., 2006a; Schouten et al., 2006b), it was recognised as a global standard. In the normal-sense orientation, the cisgene is a perfect duplicate of the endogenous gene, complete with its native promoter, introns, and terminator. According to this, cisgenesis extends the origin of the gene to the gene pool of sexually compatible species; however, there are various modifications to this these definitions (**Table 1**).

**Table 1:** Intragenesis and cisgenesis definitions by different scientists.

GOI	Regulatory sequences	Border sequence	Vector backbone	References
Cisgenics				
Full coding sequence of gene. In the presence or absence of introns	Native promoters and terminator genes from the host plant	T-DNA	Bacterial origin	(Jochemsen and Schouten, 2000)
Complete CDS containing introns derived from the recipient plant’s sexually compatible gene pool	Gene-linked coding regions	T-DNA	Bacterial origin	(Schouten et al., 2006a)
Intragenesis				
Genes with complete or incomplete coding DNA sequences (CDS) derived from a sexually compatible (recipient plant’s) gene pool. Oriented in a sense or antisense.	Promoter, spacer and terminator derived from a sexually compatible gene pool	Plant origin (P-DNA)	Bacterial origin	(Rommens, 2004)
Complete or partial CDS derived from sexually suitable genes of the recipient plant. Sense or antisense orientation	Promoter, spacer and terminator derived from a sexually compatible gene pool	P-DNA	The vector backbone from sexually suitable DNA. As an alternative, DNA mini-cycles can be used to remove the backbone prior to P-DNA transfer to the plant cell.	(Conner et al., 2010)
Complete CDS containing introns derived from the recipient plant’s sexually compatible gene pool	Promoter and terminator derived from a sexually compatible gene pool	T-DNA	Bacterial origin	(Joshi et al., 2011)

T-DNA, transfer DNA; P-DNA, plant derived DNA; CDS, complete coding sequence.

Additionally, the term “intragenesis” is used to describe situations in which P-border and vector-backbone sequences come from a DNA pool that is not sexually compatible. If future laws for cisgenic and intragenic crops are less strict than those guiding transgenic crop regulations, the meanings of cisgenesis and intragenesis may eventually need to be clarified. Because of some similarities, these kinds of genetic engineering techniques modify an organism’s DNA by utilising genetic material from within the same species or closely related species. However, there are few key differences between intragenesis and cisgenesis. When a gene is transformed by intragenesis, genetic material from the same species or a closely related species is used, but the modified gene may express itself differently than it did in the original organism. For example, a gene may be modified by adding regulatory elements to change its expression pattern or increase its level of expression. In cisgenesis, the genetic modification is performed using genetic material (coding sequence, promoter and terminator) only from the same species. The modified gene is placed back into the genome of the original organism along with its natural regulatory elements. This means that the expression pattern of the modified gene will be the same as in the original organism and limited chance of risk of unintended effects on the organism’s development or metabolism.

**Figure 1:**
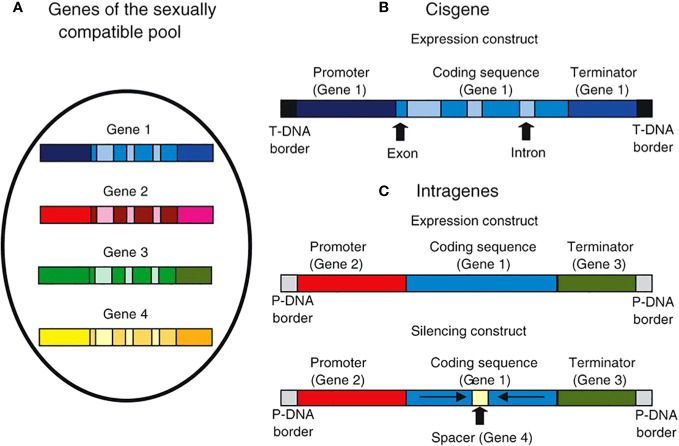
Illustration showing cisgene and intragene gene constructs. In cisgene, genes from the pool of sexually compatible individuals that include the promoter, introns, and terminator **(A, B)**. When using agrobacterium-mediated transformation, the cisgene is inserted within agrobacterium-derived T-DNA borders. Within the sexually compatible gene pool, intragenesis enables in vitro recombination of components isolated from several genes **(A, C)**. Furthermore, cDNA or gene fragments may be employed; introns are not necessary. Consequently, both silencing and expression intragenic constructions are possible (Schouten et al., 2006a).

Intragenesis and cisgenesis can overcome few of the drawbacks of conventional breeding. The breeding process can be accelerated through cisgenesis and intragenesis, which further saves time and resources compared to conventional breeding techniques. Although this transfer can also be accomplished by conventional breeding, the effectiveness and duration of these programmes depend on the crop’s duration and propagation mechanism. Additionally, the intragenesis/cisgenesis method avoids any possible “linkage drags” brought on by conventional backcross breeding. Sometimes the gene of interest is so closely related to genes responsible for inferior traits that recombination between this gene and the undesirable genetic material is nearly impossible. The intragenic and cisgenic concepts can help avoid issues with conventional breeding while improving features with limited natural allelic variability within the sexually compatible gene pool. By reintroducing a gene for traits with its own promoter and terminator (cisgenesis) or with a promoter and terminator derived from the gene pool of sexually compatible individuals (intragenesis), a higher expression level of the trait can be achieved. The expression levels can also be lowered by applying different silencing constructs (intragenesis) (**Figure 1**).

The most major barriers to implementing these practices include the possibility of potential health issues and the spread of novel genes into unrelated crop species, and also expensive, time-consuming, and protracted procedures for securing authorisation of these GM crops. With these disadvantages in mind, scientists sought a sustainable and effective solution to all of these problems, with the goal of ensuring eco-friendly crop enhancement approaches. Thus, with a pledge of environmental safety, the cisgenic technique blossomed as an alternative to the transgenic procedure. The fundamental idea behind cisgenesis is the selective utilisation of genetic material from closely related species that are capable of sexual hybridization. The gene pool that cisgenic crops enslave is the same gene pool that traditional plant breeding use (Schahczenski, 2018).

## Pre-requisites and procedures

The availability of sequence information for the genes that are to be transferred is necessary for the genetic engineering procedures known as intragenesis and cisgenesis, which transfer genes within the same species or among closely related species. For cisgenesis, the sequence information for the desired gene(s) is typically obtained from the same or a closely related plant species. Several techniques, including the plant genome’s sequencing, cDNA libraries, or PCR amplification of the desired gene, can be used to gather the information. The gene(s) can then be isolated and introduced to the target plant using standard genetic engineering techniques. Similar to cisgenesis, intragenesis normally uses the same techniques to collect the sequencing data for the desired gene(s). Cisgenesis is a genetic modification technique that involves transferring a gene of interest and regulatory elements from a closely related plant species into the target crop plant, which is necessary for proper gene expression. This results in the development of a crop plant that is genetically similar to its non-modified counterpart but with the desired trait. On the other hand, intragenesis involves the transfer of a gene of interest from the same species or a closely related species into the target plant’s genome in sense or anti-sense orientation, but without the inclusion of foreign DNA. In this technique, the gene is modified or replaced by using only DNA fragments from the same or closely related species.

After isolating a gene, it is essential to characterize it to determine its function and regulatory elements. Once the desired gene of interest and its regulatory components have been identified, vector must be used to clone them. This vector will be used to introduce the gene into the target crop plant. Transformation strategies are used to create cisgenic and transgenic plants (Schouten et al., 2006a). It has been utilised to transform *via agrobacterium-mediated* transformation through biolistic (Akhond and Machray, 2009; Lusser et al., 2012).

**Figure 2:**
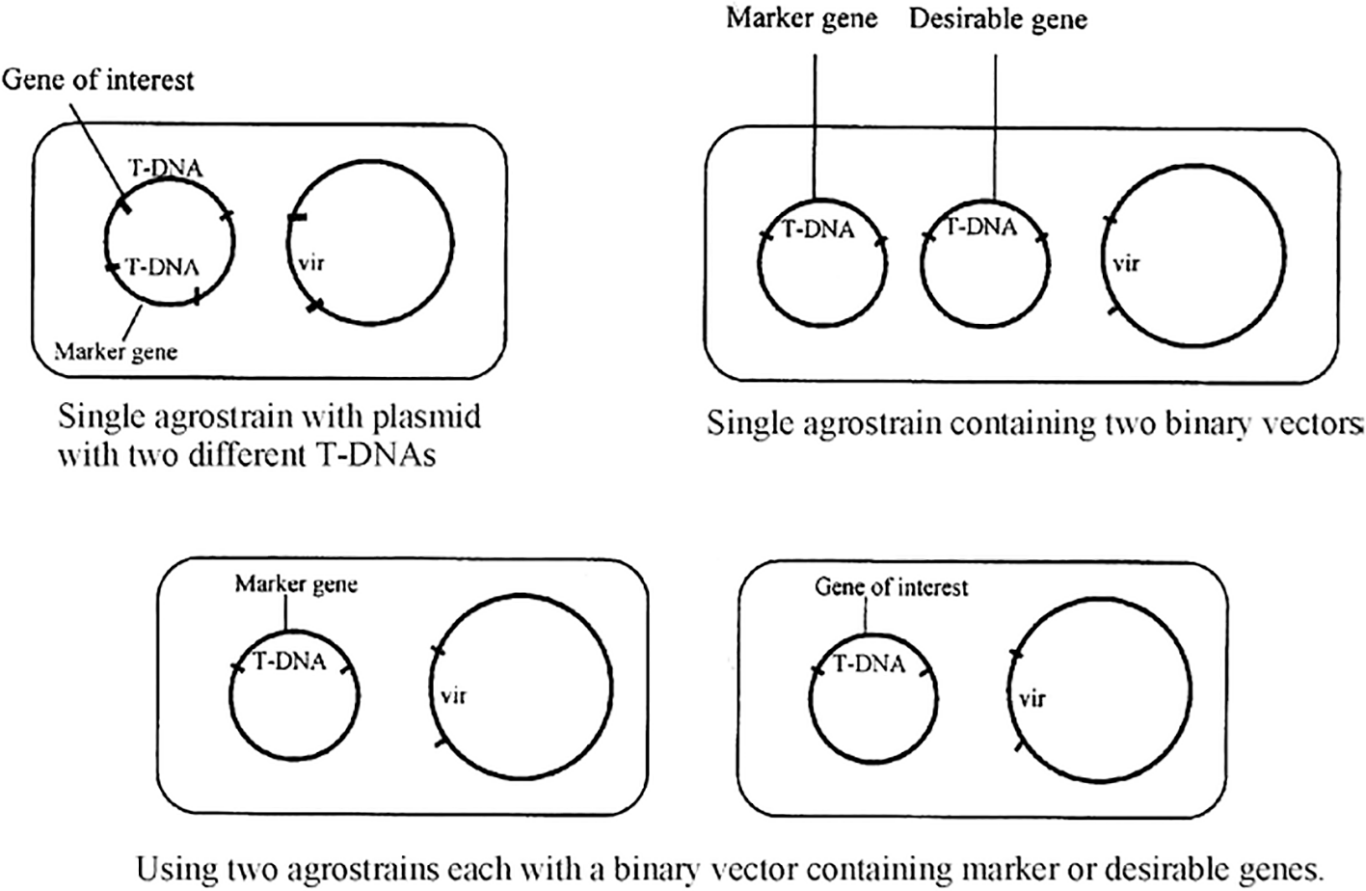
Marker elimination by co-transformation (Jaiwal et al. 2002).

To be environmentally benign, cisgenic products must be devoid of selectable marker genes and retain desirable genes from cross-compatible species. Marker gene elimination methods like co-transformation use two T-DNA regions, one with a selectable marker and another with a gene of interest. Both the T-DNAs are integrated within the same binary vector, two binary vectors within the same *Agrobacterium* or with two different *Agrobacterium* strains (**Figure 2**). Select transformants that carry both transgenes as unlinked copies of non-linked transgene loci will be separated by segregation. Although the method is believed to be efficient and mature (up to 25% of all co-transformed cell lines exhibit marker segregation), screening becomes time-consuming and expensive due to the need to examine four times as many cell lines. In recombinase-induced excision, the recombinase enzyme, which cuts two DNA recognition sequences and ligates the free ends after removing the DNA sequence in between, removes the DNA sequence coding for the selectable marker. There are three primary approaches: The Cre/lox system from bacteriophage P1 in plants is autoexcision; the Cre enzyme recognizes its specific target sites, which are *loxP* and FLP/*FRT* recombination systems from *Saccharomyces cerevisiae*; the FLP recombinase acts on the *FRT* sites; and the R/*RS* recombination system from *Zygosaccharomyces rouxii*, where R and *RS* are the recombinase and recombination sites, respectively (Zuo et al., 2001).

To produce cisgenic or almost cisgenic plants, several techniques can be used, including genome editing tools, homologous recombination of desired genetic material and A*grobacterium*-mediated transformation of constructs containing cisgenic DNA (Hou et al., 2014). Compared to other methods of genome editing, such as chimeric DNA/RNA gene repair oligonucleotides (Gamper et al., 2000), zinc-finger nucleases (Townsend et al., 2009), homing endonucleases (Hafez and Hausner, 2012), transcription activator-like effector nucleases (TALENs) (Bogdanove and Voytas, 2011) and clustered regularly interspaced short palindromic repeats (CRISPRs). With the help of traditional *Agrobacterium*-mediated transformation methods, stable transgenic plants are created. Such processes can produce plants with T-DNA boundaries obtained from bacteria, which may damage a gene or regulatory DNA at the insertion location.

While the occurrence of a native homologue and a cisgene in the same plant is possible when a cisgene is randomly inserted into the genome (Schouten et al., 2006a). When compared to Agrobacterium-mediated transformation systems, the CRISPR/Cas9 gene replacement (gene knock-in) technique has inherent advantages because it enables the control of transgene copy number, the introduction of only cisgenic material, the ability to remove native genes and replace them with cisgenes, and the ability to perform site-specific genome integration events. It may be possible to alter the level, timing and mode of expression of a certain gene or collection of genes by substituting native regulatory regions. Additionally, this method can be used to add, modify, or replace certain genes or parts of gene, such as regulatory regions, introns, exons and targeting signals or even SNPs.

## Applications and approaches: improving processing qualities, disease resistance (biotic and abiotic stress) of various crops

### Apple

Majority of today’s commercial apple varieties are vulnerable to scab, the most harmful fungus that affects commercial apples (Joshi et al., 2011), caused by *Venturia inaequalis*. The Vf locus (*Rvi6* gene) from the wild apple *Malus floribunda* 821 is the resistance source that is most frequently employed in traditional breeding of apples (Szankowski et al., 2009). Although the resistance gene is successfully transferred to the elite cultivars, the process takes a very lengthy time and is linked to undesirable linkage drag. In this regard, an effort was made to develop scab-resistant cisgenic apples. The *Rvi6* gene, along with its own promoter and terminator, were initially transferred into apple cv. Gala in order to create a cisgenic apple with resistance to scab (Vanblaere et al., 2011). Parallel to this, intragenic apple lines were developed (Vanblaere et al., 2014) resistant to scab by transferring *Rvi6* gene driven by the rubisco gene’s promoter and terminator. Scab resistance of these two lines (cisgenics and intragenics) was assessed in the greenhouse and field. The resistance levels in the field proved to concur with the resistance levels in the greenhouse (Krens et al., 2015). The suitability of a visual selection approach using MdMYB10 for creating transgenic apples was reported (Kortstee et al., 2011). Gene cassette was constructed, *MdMYB10* gene, flanked by its native promoter and terminator and included it within the T-DNA borders of the minimal vector (Krens et al., 2015). They also described the selection of cisgenic “Gala” and “Jumami” lines purely on the basis of anthocyanin production and red staining of calluses and plant shoots. This selection method gave an idea of using *MYB10* as a selectable marker in the development of cisgenic and intragenic apples by combining it with a trait of interest. The development and selection of new fire blight-resistant apple genotypes would significantly enhance the control of this deadly *Erwinia amylovora* disease. Currently, conventional breeding is used to create such resistant genotypes, but innovative breeding technologies like cisgenesis could represent a different strategy. In this direction, a cisgene apple line (C44.4.146) was developed using *FB_MR5* gene from the wild species *Malus × robusta 5* (Mr5), which confers fire blight resistance by transferring into Gala Galaxy by employing *A. tumefaciens*- mediated transformation using a binary vector. No transgenes were found in the line C44.4.146, except the cisgene *FB_MR5*, which is regulated by its native regulatory sequences (Kost et al., 2015).

### Potato

The majority of potatoes processed for human consumption go into making French fries and potato (*Solanum tuberosum*) chips. Consequently, the potato types processing characteristics are of the highest priority, and the major problem in the processing industries is the enzymatic browning of potatoes. Potatoes become brown enzymatically due to polyphenol oxidases (PPOs) leaking from broken plastids. Black melanin then precipitates due to the cytoplasmic oxidation of polyphenols, which degrades the quality of the tubers over time. With the use of a silencing construct regulated by the GBSS promoter and the Ubi-3 terminator and made entirely of potato DNA, a *Ppo* gene that is mainly expressed in mature tubers was rendered inactive. Between potato P-DNA border sequences, this gene cassette was introduced. The resulting intragenic potatoes have greater black spot bruise tolerance and less browning in the tubers. A multigene intragenic silencing construct with portions of the *Ppo*, *R1*, and *PhL* genes in sense and antisense orientations were employed (Rommens et al., 2006). The resulting intragenic potato lines generated tubers with noticeably less glucose and fructose buildup and less browning. It was anticipated that lower tuber glucose and fructose levels would result in less acrylamide production during the French fry processing of the tubers. When asparagine and the carbonyl group of reducing sugars interact during heating, acrylamide is the result. Acrylamide has been linked to the production of several degenerative disorders, including cancer, when taken in large quantities. The quantity of acrylamide was significantly reduced (30%) in french fries made from intragenic tubers with low levels of glucose and fructose (Rommens et al., 2006). Silencing the genes involved in asparagine production is an alternate method for producing minimal acrylamide French fries. Asparagine (ASN) makes up to 25% of the total free amino acid pool in potato tubers and is the major free amino acid. Asparagine synthetase (*Ast*) catalyses the last step in ASN formation. High tuber concentrations of ASN are undesirable because they can oxidise into the carcinogenic chemical acrylamide while heating starchy meals in a low-water setting. Acrylamide and glycidamide, its reactive metabolites, are both neurotoxins and perhaps carcinogens. An intriguing new plant breeding technology idea for gene silencing is intragenesis. To illustrate, the asparagine synthase-1 (*StAst1 and StAst2*) gene was silenced in potatoes using the intragenesis idea to lessen the production of acrylamide in potatoes during baking and frying. The gene was driven by a tuber-specific GBSS promoter. Only 5% of the acrylamide levels found in Ast silenced potato lines, but under field conditions, tubers that developed were tiny and fractured. Interestingly, silencing only the *StAst1* gene produced intragenic potatoes with 70% lower acrylamide levels after processing and a normal tuber phenotype in field experiments (Chawla et al., 2012). Intragenic model is not only adopted for gene silencing but also adapted to enhance specific protein expression. In this view, maize Rubisco activase (*Rca*) gene was successfully over-expressed intragenically in corn (Almeraya and Sánchez-de-Jiménez, 2016). This demonstrated the feasibility of maize intragenic modification for improving crop yield, retaining the plant genome free of foreign DNA and gaining significant additional time and labour savings.

### Barley

The conversion of stored phytic acid, which makes up around 70% of the total phosphate in seeds, into bioavailable phosphate. Phytases are able to liberate this phosphate enzymatically. However, there is little endogenous phytase activity in mature barley grains. The majority of the phytic acid in barley grain is secreted, dispersed by manure, and eventually ends up in the water, where it promotes the growth of algae and eutrophication. Because of this, when barley grains are used as animal feed, only about 30% of the phosphate is actually absorbed by the animals. The remaining 70% is instead expelled in faeces and urine and released when the manure is spread on fields, damaging the aquatic environment. The phytase gene (*HvPAPhy_a*), which is predominantly expressed during seed development and is accountable for the preformed phytase in the mature grain, was inserted into barley with its native promoter and terminator as part of the cisgenic method (Holme et al., 2012). In addition, three PAP07, PAP05, and PAP03 barley lines (Holme et al., 2012) had the *HvPAPhy*_a cisgene (single copy) introduced into them. *HvPAPhy_a* cisgenes were stacked by crossing and double haploid production from progenies of PAP05, PAP05 and PAP03 carrying single copies. Stacked lines displayed a clear and linear increase in mature grain phytase activity (Holme et al., 2020). Nitrogen (N) is crucial for plant development and a major factor in crop production. Plants primarily absorb nitrogen in ammonical form ( ${\text{NH}}_{4}^{+}$ ). The condensation of ${\text{NH}}_{4}^{+}$ and glutamate is then catalysed by glutamine synthetase (GS), constituting the initial step in the biosynthesis of organic N molecules. GS1 participates in the basic ${\text{NH}}_{4}^{+}$ absorption process in the roots and is also engaged in the assimilation and recycling of ${\text{NH}}_{4}^{+}$ that is produced during senescence from the breakdown of proteins. Consistent promoters have been employed to overexpress GS1 in an attempt to boost plant growth and Nitrogen use efficiency (NUE) (Thomsen et al., 2014; Seger et al., 2015; Urriola and Rathore, 2015; Lu et al., 2018) but have shown inconsistent results. This unpredictability may be caused by unwanted pleiotropic effects from the usage of constitutive promoters or post-translational regulation altering enzyme activity. An attempt was made to over express the *HvGS1* gene with indigenous promoter in barley (Gao et al., 2018). In comparison to wild-type plants, additional copies of native *HvGS1*cisgenic lines in barley had higher GS1 enzyme activity, higher grain yields, and higher NUE when grown under three different N sources and two levels of ambient CO_2_. When plants were exposed to increased (800-900 lL/L) ambient CO_2_, the grain protein concentration in the GS1 over-expressing lines did not decrease, in contrast to the wild-type.

### Lucerne

The high quantities of the indigestible fibre component, lignin, reduce the feed quality of Lucerne. Transformation with intragenic development silencing the caffeic acid o-methyltransferase gene (*Comt*) resulted in intragenic lucerne with lower amounts of lignin (Weeks et al., 2008). This design makes use of the Lucerne plastocyanin promoter (PetE). For silence, two segments of the natural caffeic acid 0-methyltransferase gene were introduced as inverted repetitions between two convergently oriented PetE promoters. The resulting intragenic Lucerne had lower amounts of lignin and thus superior feed quality (Liu and Whittier, 1995).

### Grapes

Regulatory sequence ignorance limits cisgenesis and intragenesis as alternatives to transgenesis to a few species. Full genomic sequences allow genes and their natural regulatory areas to improve crop traits. Despite its global economic importance, no cisgenic or intragenic grapevine plants have been described. Vintners worry about transgenesis, which brings foreign genes into prime grape varieties and influences wine characteristics. Thus, transgenic technology improves grape quality by studying native genes suitable for genetic change and characterising promoter expression, which is more important than identifying them. Testing numerous grape promoters and approaches has found regulatory sequences with diverse expression patterns and genes expressed just during ripening, in response to sugars, senescence and biotic stress. As mesocarp cells absorb sugars during veraison, *Vitis vinifera* berries expand and mature (Deluc et al., 2007). During ripening, sugar accumulation produces phenolic chemicals, anthocyanin, and precursors to aromas (Agasse et al., 2009). Berry glucose and fructose are converted into anthocyanin in cell cultures and fruit discs (Larronde et al., 1998; Zheng et al., 2009). This is caused by structural gene transcription factors in the anthocyanin pathway. To synthesise anthocyanin, UFGT is controlled by VvMYBA1, a member of the R2R3MYB family (Kobayashi et al., 2002; Walker et al., 2007; Cutanada et al., 2009). During berry development, soluble sugars increase VvMYBA1 transcript levels and monosaccharides build up in the vacuole of the cell after sugar enters it via sugar transporters (Williams et al., 2000; Agasse et al., 2009). The cloning of the grape monosaccharide transporter VvHT1-6 has been done. Six putative monosaccharide transporters called VvHT1-6 have been cloned in grapes (Fillion et al., 1999; Vignault et al., 2005; Hayes et al., 2007). The majority of them are found in the cell membrane. Less is known, however, about monosaccharide transporters found in the vacuole membrane or tonoplast. The VvHT6 gene has been identified. The predicted protein has a central loop, which is typical of arabidopsis tonoplast transporter proteins (Wormit et al., 2006). VvHT6 was thus renamed VvTMT2 (*Vitis vinifera*) Tonoplast Monosaccharide Transporter 2). VvTMT2 expression during berry ripening peaks at veraison and declined during the ripening stage. This pattern of expression corresponds to an rise in berry sugar content. In silico investigations of the VvMYBA1 and VvTMT2 promoters using the GRAPE-Hunt programme reveal the existence of cis regions associated with sugar regulation, implying that both genes’ expression may be regulated by sugars. VvMYBA1 and VvTMT2 are two genes that are expressed during grape ripening. This type of regulation allows these promoters to be used to express genes at specific periods throughout berry development, reducing the possibility of affecting other features of berry maturity.

### Durum wheat

In durum wheat, biolistic transformation was conducted to enhance bread-making properties. Wheat D genome genes encoding the 1D × 5 and 1Dy10 glutenin subunits with their own native endosperm promoters and terminators were cloned and transferred. As a positive selectable marker, *E*. *coli* phosphomannose isomerase (pmi) was removed from minimal gene cassettes under genetic segregation and positive selection (Gadaleta et al., 2008).

### Poplar trees

In poplar trees, the effects on plant growth rate and wood properties from the insertion of five cisgenes (PtGA20ox7, PtGA2ox2,Pt RGL1_1, PtRGL1_2 and PtGAI1) from Populus trichocarpa clone Nisqually-1 that encode proteins for gibberellin metabolism or signalling into Populus Tremula × alba (clone INRA 717-1B4) have been studied (Han et al., 2011). Cisgenic plants expressing PtGA20ox7 showed increased shoot regeneration in vitro, accelerated early growth. PtRGL1_1 and PtGA2ox2 caused reduced growth, while PtRGL1_2 gave rise to plants that grew normally but had significantly longer xylem fibres. PtGAI1 and PtGA20ox7 gave rise to increased variance among events for early diameter and volume index.

A case-by-case account of several crops that are now being developed or are in the process of being developed through intragenesis or cisgenesis is provided in the below section and field-tested intragenic and cisgenic crops and the status of their regulation at the time are each covered in their own section. To date, intragenesis and cisgenesis have altered a number of distinct features in a variety of crops (**Table 2**).

**Table 2:** Crop improvement through cisgenesis/intragenesis.

Cisgenesis				
Crop	Approach	Trait	Gene	References
Potato	Expression and Gene stacking	Late blight resistance	R-genes	(Haverkort et al., 2009; Haverkort et al., 2016)
Barley	Overexpression	HvPAPhy_a	Improved grain phytase activity	(Holme et al., 2012)
	Overexpression	Nitrogen use efficiency	Glutamine synthetase (GS1)	(Gao et al., 2018)
Durum wheat	Expression	Improved baking quality	*1Dy10*	(Gadaleta et al., 2008)
Apple	Overexpression	Fire blight-resistant	*FB_MR5*	(Kost et al., 2015)
Intragenesis				
Potato	Silencing	High amylopectin	GBSS	(de Vetten and Wolters, 2003)
		Limit acrylamide in French Fries	*StAs1,StAS2* and *StAs1*	(Rommens et al., 2008; Chawla et al., 2012)
Apple	Expression	Scab resistance	*HcrVf2*	(Conner et al., 2010)
Strawberry	Over expression	Grey mould resistance	*PGIP* (Polygalacturonase Inhibiting Protein)	(Schaart et al., 2004)
Corn	Over expression	Check to see if the increment response is similar to the levels of Rca expression attained via traditional selection.	*ZmRca* (Maize Rubisco Activase)	(Almeraya and Sánchez-de-Jiménez, 2016)

## Advantages and disadvantages

Compared to conventional plant breeding, the new breeding technologies of cisgenesis and intragenesis have a variety of benefits. By inserting genes from the conventional breeding pool, the cisgenesis approach enhances genetic modification through higher accuracy, pace and complexity of trait improvement (Jo, 2013). Cisgenesis is the capacity to rapidly and precisely modify an existing cultivar’s genetic makeup without encountering any linkage drag issues (Telem et al., 2013). Linkage drag is the issue that arises when a gene of interest is linked to other unwanted genes, often harmful ones they are being introduced into the genome in an effort to improve it (Delwaide, 2014). In the sexually compatible gene pool, traits with minimal allelic dissimilarity and variation can be altered via cisgenic breeding (Schaart et al., 2016). Additionally, it is possible to develop cultivars resistant to disease with clear economic and environmental benefits by using genes for abiotic and biotic stress resistance from a crossable donor (Vanblaere et al., 2014). Additionally, cisgenes and intragenes essentially exploit the gene pool of the breeders. Natural genes are called cisgenes, and intragenes are the functional parts of cisgenes that come from the crop plant itself or from sexually compatible species. Cisgenesis, which essentially entails a linkage drag-free introgression breeding process in a single step, combines the applications of cisgenes with marker-free transformation. Food safety is yet another prized benefit of cisgenesis. For instance, to restore diverse glycoalkaloids lost via breeding, wild species of *Solanum* were exploited as a source of genetic diversity (Van Gelder, 1989). Since wild species can serve as a source of genetic variation for the reintroduction of advantageous compounds that have been eliminated as a result of domestication and breeding (Jacobsen and Schouten, 2008).

Cisgenesis does have multiple risks and inconveniences, including an unidentified insertion site and an insertion site mutation. Because cisgenes are randomly inserted into the plant genome, which could result in unexpected risks. In fact, normal plant breeding also results in the occurrence of random alien DNA insertions into the plant genome. Additionally, this arbitrary insertion of one or more genes into the genome is vulnerable to the impact of nearby genes on expression or the opposite. Contrastingly, it underlines the possibility that cisgenic breeding might still result in the addition of unique features to the cisgenic product and as a result, create new dangers. Another potential downside of cisgenesis is the cisgene’s insertion site mutation and the unanticipated phenotypic changes (Jacobsen and Schouten, 2008).

## Safety issues

In most nations, cisgenic or intragenic crops are regulated like transgenic crops. Cisgenic plants are more widely accepted than any other genetic alterations, according to Europe, North America, Japan, Australia, and New Zealand. Cisgenic plants are more strictly regulated in Australia, Canada, and the US than in Europe, Japan, and New Zealand. These nations have a variety of public perceptions and contributing factors that affect consumer choices. In contrast to North Americans, who are more concerned with product quality and health, Europeans are more inclined to adopt cisgenic plants and their derived products if they have an environmental advantage. In contrast, New Zealand debates the influence of cisgenic plants on linked businesses like meat export and tourism (Daye et al., 2023).

Directive 2001/18/EC governs the purposeful deployment of GMO crops in the EU. However, non-recombinant nucleic acid procedures, such as mutagenesis and “cell fusion, including protoplast fusion of plant cells of organisms that can exchange genetic material through conventional breeding methods,”are exempt from the regulation according to Annex IB. The three Dutch researchers (Schouten, Jacobsen and Krens) who introduced the cisgenesis concept in 2006, have advocated for cisgenesis-derived plants to be included in Annex IB and exempt from regulation (Jacobsen and Schouten, 2009; Schouten et al., 2006a). In 2007, the European Commission (EC) formed the Novel Techniques Working Group (NTWG) to assess novel breeding methods and determine if they were genetic modifications. Zinc finger nucleases, oligonucleotide directed mutagenesis, RNA-dependent DNA methylation, grafting on GM root stock, reverse breeding and agro-infiltration were among the seven plant enhancement methods. After that, the EC asked the European Commission Joint Research Centre for Prospective Technological Studies to rank these novel molecular tools in plant breeding. Intragenesis/cisgenesis scored first and second among the seven innovative techniques in the scientific articles and patents (Lusser et al., 2012).

According to the EFSA panel, intragenic and cisgenic plants should adhere to current GM advice for food, feed, safety and environmental risk assessments. As per the information regarding the nature, features and history of safe usage, the amount of risk assessment data required for these plants can be decreased on a case-by-case basis. Contrarily, cisgenic and intragenic crops are currently regulated in the United States just like transgenic crops. The U.S. Department of Agriculture (USDA) would still need to approve the release of cisgenic crops modified with other traits into the environment. Although the USDA was provided with the EPA’s proposal for a cisgenic crop exemption, the latter has yet to issue a statement to that effect. The French High Council of Biotechnologies (HCB), EFSA, and the Scientific Advice Mechanism in Europe have found that plants created via targeted mutagenesis, cisgenesis, and intragenesis technologies have no health or environmental impacts. EFSA scientific opinions on SDN-1, SDN-2, ODM, cisgenesis, and intragenesis showed no dangers compared to standard breeding procedures. The assessment of cisgenic and intragenic plants using new genetic techniques (NGTs) may require fewer assessments as the extra genetic material is integrated in a site-directed manner. If the donor plant has been used safely as food or feed in earlier times, certain aspects of the comparison analysis, like toxicity, allergenicity and nutritional value, may not be essential. As far as environmental risk assessment is concerned, all of the factors listed in current regulations can be used for both cisgenic and intragenic plants. As a result, the GMO panel considers that the current criteria are only partially applicable and adequate. A smaller amount of data may be required for risk assessment of cisgenic or intragenic plants collected using NGTs on a case-by-case basis. Genome-edited crops are free from exogenous gene/DNA. So the resultant genome edited crops are exempted, similar to those of SDN1 and SDN2, under the provisions of Rules 7 to 11 (of the Rules 1989) of EPA, 1986. SDN1, SDN2 and ODM scientific concepts are from 2020, whereas a cisgenesis/intragenesis opinion is from 2012. *Agrobacterium-mediated* transformation and direct gene transfer were available at that time, although several of the issues are not linked to a specific technology. In addition to established genomic techniques (EGTs), genome editing methods like SDN can now make cisgenic and intragenic organisms alone or in combination. In light of this, the commission would ask EFSA to confirm whether the considerations and conclusions of the 2012 EFSA scientific opinion on cisgenesis/intragenesis are still relevant. WGG and AFBV recommend exempting under fourth category of modified cisgenic plants from the GMO regulatory framework, utilising the exemption mechanism in Article 3a (Jochemsen and Schouten, 2000; Rommens, 2004; Schouten et al., 2006a; Schouten et al., 2006b) of Directive 2001/18/EC (Annex I B/IC) ([[NoAuthor]]; Ewen et al., 2022; Proposal by AFBV and WGG for amendments to GMO legislation, 2022).

## Conclusion

Intragenesis and cisgenesis use gene bearing the same gene pool asin conventional breeding. Thus, cisgenesis and intragenesis could be handled like normal breeding and can be excluded from transgenic regulations. Several surveys and focus group interviews in the US and Europe show that intragenic and cisgenic crops are more allowable than transgenic crops, encouraging their use to produce them. Less stringent transgenic agricultural regulations might boost commercial use. Ectopic genome insertion in cisgenesis and intragenesis may create unforeseen pleiotropic effects. The recipient plant retains its endogenous gene. For site-specific mutagenesis, gene targeting must be prioritised. Cisgenesis and intragenesis have opened a fresh discourse between scientists, breeders, and consumers about consumer-friendly genetically modified crops. However, compared to transgenic crops, intragenic/cisgenic crops will require less stringent regulations. Through these approaches, cisgenesis, intragenesis, and ways for creating marker-free plants can be used as efficient alternatives to introduce and domesticate agriculturally significant genes in enhanced current varieties in a single step. New parents can also be created for future crossings to create new kinds. Future progress in creating and commercialising cisgenic and intragenic crops will be dependent on global readiness to apply less stringent regulation to these crops. In order to obtain an extra tool for crop improvement and increase the number of cis/intragenic crops developed, breeders in small-scale breeding and seed businesses need help, such as less severe regulation of cis/intragenic crops and cheaper approval costs. In recent years, efforts to modify plants have been made using constructs constructed solely from DNA sequences originating from the same or sexually compatible plant species, known as cisgenics. In the future, it is anticipated that the public would accept cisgenic plants without hesitation or resistance, and the technology will support breeding initiatives aimed at introducing desirable features that were lost during the domestication of wild crop species.

## Author contributions

VS: Supervision, Investigation, Writing – review & editing. PK: Conceptualization, Writing – original draft, Supervision. TR: Conceptualization, Writing – original draft. DS: Investigation, Writing – review & editing.

## References

[B1] Agasse A. Vignault C. Kappel C. Conde C. Geros H. Delrot S. (2009). “Sugar transport and sugar sensing in grape,” in Grapevine molecular physiology and biotechnology. Ed. Roubelakis-Angelakis K. A. (New York: Springer), 105–139. doi: 10.1007/978-90-481-2305-6_5

[B2] Akhond M. A. Y. Machray G. C. (2009). Biotech crops: technologies, achievements and prospects. Euphytica 166, 47–59. doi: 10.1007/s10681-008-9823-1

[B3] Almeraya E. V. Sánchez-de-Jiménez E. (2016). Intragenic modification of maize. J. @ Biotechnol. 238, 35–41. doi: 10.1016/j.jbiotec.2016.09.009 27641689

[B4] (2022). Guidelines for the safety assessment of Genome edited plants, Department of Biotechnology, Ministry of Science and Technology (Government of India), 4–5.

[B5] Bogdanove A. J. Voytas D. F. (2011). TAL effectors: customizable proteins for DNA targeting. Science 333, 1843–1846. doi: 10.1126/Science.1204094 21960622

[B6] Chawla R. Shakya R. Rommens C. M. (2012). Tuber-specific silencing of asparagine synthetase-1 reduces the acrylamide-forming potential of potatoes grown in the field without affecting tuber shape and yield. Plant Biotechnol. J. 10, 913–924. doi: 10.1111/j.1467-7652.2012.00720 22726556

[B7] Conner A. Pringle J. Lokerse A. Jacobs J. Barrell P. Deroles S.. (2010). Plant transformation using DNA minicycles. WO patent Appl.

[B8] Cutanada M. Ageorges A. Gomez C. Vialet S. Terrier N. Romieu C Torregrosa L. (2009). Ectopic expression of *VlmybA1* in grapevine activates a narrow set of genes involved in anthocyanin synthesis and transport. Plant Mol. Biol. 69 (6), 633–648. doi: 10.1007/s11103-008-9446-x 19096760

[B9] Daye C. Spok A. Allan A. C. Yamaguchi T. Sprink T. (2023). Social acceptability of cisgenic plants: public perception, consumer preferences, and legal regulation (Springer), 43–75.

[B10] Deluc L. G. Grimplet J. Wheatley M. D. Tillett R. L. Quilici D. R. Osborne C.. (2007). Transcriptomic and metabolite analyses of Cabernet Sauvignon grape berry development. BMC Genomics 8, 429. doi: 10.1186/1471-2164-8-429 18034876 PMC2220006

[B11] Delwaide A. C. (2014). “European consumer’s attitudes towards cisgenic rice,” in IMRD Consortium.

[B12] de Vetten N. Wolters A. (2003). Raemakers K A transformation method for obtaining markerfree plants of a cross-pollinating and vegetatively propagated crop. Nat. Biotechnol. 21 (4), 439–442. doi: 10.1038/nbt801 12627169

[B13] Ewen M. Jean-Louis B. Tamas D. Ian C. D. Michelle M. E. Leslie G. F.. (2022). Updated scientific opinion on plants developed through cisgensis and intragenesis. eJ EFSA 20 (10), 7621. 10.2903/j.efsa.2022.7621 PMC9583739 36274982

[B14] Fillion L. Ageorges A. Picaud S. Coutos-Thevenot P. Lemoine R. Romieu C.. (1999). Cloning and expression of a hexose transporter gene expressed during the ripening of grape berry. Plant Physiol. 120 (4), 1083–1094. doi: 10.1104/pp.120.4.1083 10444092 PMC59342

[B15] Gadaleta A. Giancaspro A. Blechl A. E. Blanco A. (2008). A transgenic durum wheat line that is free of marker genes and expresses 1Dy10. J. Cereal Sci. 48 (2), 439–445. doi: 10.1016/j.jcs.2007.11.005

[B16] Gamper H. B. Parekh H. Rice M. C. Bruner M. Youkey H. Kmiec E. B.. (2000). The DNA strand of chimeric RNA/DNA oligonucleotides can direct gene repair/conversion activity in mammalia and plant cell-free extracts. Nucleic Acids Res. 28, 4332–4339. doi: 10.1093/nar/28.21.4332 11058133 PMC113138

[B17] Gao Y. de Bang T. C. Schjoerring J. K. (2018). Cisgenic overexpression of cytosolic glutamine synthetase improves nitrogen utilization efficiency in barley and prevents grain protein decline under elevated C02. J. Plant Biotechnol. 17, 1209–1221. doi: 10.1111/pbi.13046 PMC6576097 30525274

[B18] Hafez M. Hausner G. (2012). Homing endonucleases: DNA scissors on a mission. Genome 55 (8), 553–569. doi: 10.1139/g2012-049 22891613

[B19] Han K. M. Dharmawardhana P. Arias R. S. (2011). Gibberellin-associated cisgenes modify growth, stature and wood properties in. Populus. Plant Biotechnol. J. 9 (2), 162–178. doi: 10.1111/j.1467-7652.2010.00537.x 20573046

[B20] Haverkort A. J. Boonekamp P. M. Hutten R. Jacobsen E. Lotz L. A. P. Kessel G. J. T.. (2016). Durable late blight resistance in potato through dynamic varieties obtained by cisgenesis: Scientific and societal advances in the DuRPh project. Potato Res. 59, 35–66. doi: 10.1007/s11540-015-9312-6

[B21] Haverkort A. J. Struik P. C. Visser R. G. F. Jacobsen E. (2009). Applied biotechnology to combat late blight in potato caused by. Phytophthora infestans. Potato Res. 52, 249–264. doi: 10.1007/s11540-009-9136-3

[B22] Hayes M. A. Davies C. Dry I. (2007). Isolation, functional characterization, and expression analysis of grapevine (*Vitis vinifera* L.) hexose transporters: diff erential roles in sink and source tissues. J. Exp. Bot. 58 (8), 1985–1997. doi: 10.1093/jxb/erm061 17452752

[B23] Holme I. B. Dionisio G. Brinch-Pedersen H. Wendt T. Madsen C. K. Vincze E.. (2012). Cisgenic barley with improved phytase activity. J. Plant Biotechnol. 10, 237–247. doi: 10.1111/pbi.12055 21955685

[B24] Holme I. B. Madsen C. K. Wendt T. Brinch-Pedersen H. (2020). Cisgenes in barley is a potent strategy for increasing mature grain phytase activity. Front. Plant Sci. 11. doi: 10.3389/fpls.2020.592139 PMC7644513 33193549

[B25] Hou H. Atlihan N. Lu Z. X. (2014). New biotechnology enhances the application of cisgenesis in plant breeding. Front. Plant Sci. 5. doi: 10.3389/fpls.2014.00389 PMC4127943 25157261

[B26] Jacobsen E. Schouten H. J. (2008). Cisgenesis, a new tool for traditional plant breeding, should be exempted from the regulation on genetically modified organisms in a step by step approach. Potato Res. 51 (1), 75–88. doi: 10.1007/s11540-008-9097-y

[B27] Jaiwal P. K. Sahoo L. Singh R. P. (2002). Strategies to deal with the concern about marker genes in transgenic plants: Some environment friendly approaches. Curr. Sci. 83 (2), 128–136. https://www.jstor.org/stable/24106215

[B28] Jo K. R. (2013). Unveiling and deploying durability of late blight resistance in potato from natural stacking to cisgenic stacking. Doctoral dissertation Wageningen Univ.

[B29] Jochemsen H. Schouten H. J. (2000). “Ethische beoordeling van genetische modificatie,” in Toetsen en Begrenzen. Een Ethische en Politieke Beoordeling van de Moderne Biotechnologie. Ed. Jochemsen H. (Amsterdam, Netherlands: Buijten and Schipperheijn), 88–95.

[B30] Joshi S. G. Schaart J. G. Groenwold R. Jacobsen E. Schouten H. J. Krens F. A. (2011). Functional analysis and expression profiling of HcrVf1 and HcrVf2 for development of scab resistant cisgenic and intragenic apples. Plant Mol. Biol. 75, 579–591. doi: 10.1007/s11103-011-9749-1 21293908 PMC3057008

[B31] Kobayashi S. Ishimaru M. Hiraoka K. Honda C. (2002). Myb-related genes of the Kyoho grape (*Vitis labruscana*) regulate anthocyanin biosynthesis. Planta 215, 924–933. doi: 10.1007/s00425-002-0830-5 12355152

[B32] Kortstee A. J. Khan S. A. Helderman C. Trindade L. M. Wu Y. Visser R. G. F. (2011). Anthocyanin production as a potential visual selection marker during plant transformation. Transgenic Res. 20, 1253–1264. doi: 10.1007/s11248-011-9490-1 21340526 PMC3210953

[B33] Kost T. D. Gessler C. Jansch M. Flacjowsky H. Patocchi A. Bronggini G. A. L. (2015). Development of the first cisgenic apple with increased resistance to fire blight. PloS One 10 (12), e0143980. doi: 10.1371/journal.pone.0143980 26624292 PMC4666654

[B34] Krens F. A. Schaart J. G. van der Burgh A. M. Tinnenbroek-Capel I. E. M. Groenwold R. Kodde L. P.. (2015). Cisgenic apple tree; development, characterization and performance. Forntiers Plant Sci. 6 (286). doi: 10.3389/fpls.2015.00286 PMC4410516 25964793

[B35] Larronde F. Krisa S. Decendit A. Chèze C. Deffieux G. Merillon J. M. (1998). Regulation of polyphenol production in Vitis vinifera cell suspension cultures by sugars. Plant Cell Rep. 17, 946–950. doi: 10.1007/s002990050515 30736545

[B36] Liu Y. G. Whittier R. F. (1995). Thermal asymmetric interlaced PCR: automatable amplification and sequencing of insert end fragments from P1 and YAC clones for chromosome walking. Genomics 25, 674–681. doi: 10.1016/0888-7543(95)80010-J 7759102

[B37] Lu T. Liu L. Wei M. Liu Y. Qu Z. Yang C.. (2018). The effect of poplar PsnGS1.2 overexpression on growth, secondary cell wall, and fiber characteristics in tobacco. Front. Plant Sci. 9. doi: 10.3389/fpls.2018.00009 PMC5780347 29403519

[B38] Lusser M. Parisi C. Plan D. Rodríguez-Cerezo E. (2012). Deployment of new biotechnologies in plant breeding. Nat. Biotechnol. 30 (3), 231–239. doi: 10.1038/nbt.2142 22398616 PMC7097357

[B39] Proposal by AFBV WGG for amendments to GMO legislation (2022) Draft Amendment to EC Directive 2001/18 based on Netherlands 2017 proposal. Available at: https://www.biotechnologies-vegetales.com/wp-content/uploads/2022/07/AFBV-WGG-Amendment-revised-6-July-2022.pdf.

[B40] Rommens C. M. (2004). All-native DNA transformation: a new approach to plant genetic engineering. Trends Plant Sci. 9, 457–464. doi: 10.1016/j.tplants.2004.07.001 15337496

[B41] Rommens C. M. Yan H. Swords K. Richael C. Ye J. (2008). Low acrylamide French fries and potato chips. Plant Biotechnol. 6 (8), 843–853. doi: 10.1111/j.1467-7652.2008.00363.x PMC2607532 18662372

[B42] Rommens C. M. Ye J. Richael C. Swords K. (2006). Improving potato storage and processing characteristics through all-native DNA transformation. J. Agric. Food Chem. 54, 9882–9887. doi: 10.1021/jf062477l 17177515

[B43] Schaart J. G. Krens F. A. Pelgrom K. T. B. Mendes O. Rouwendal G. J. A. (2004). Effective production of marker-free transgenic strawberry plants using inducible site-specific recombination and a bifunctional selectable marker gene. Plant Biotechnol. J. 2 (3), 233–240. doi: 10.1111/j.1467-7652.2004.00067.x 17147614

[B44] Schaart J. G. van de Wiel C. C. M. Lotz L. A. P. Smulders M. J. M. (2016). Opportunities for products of new plant breeding techniques. Trends Plant Sci. 21 (5), 438–449. doi: 10.1016/j.tplants.2015.11.006 26654659

[B45] Schahczenski J. (2018). Genetically modified crops: transgenics and cisgenics, NCAT agril. Nat. Res. 189, 1–19.

[B46] Schouten H. J. Krens F. A. Jacobsen E. (2006b). Do cisgenic plants warrant less stringent oversight. Nat. Biotechnol. 24, 753. doi: 10.1038/nbt0706- 16841052

[B47] Schouten H. J. Krens F. A. Jacobsen E.. (2006a). Cisgenic plants are similar to traditionally bred plants. EMBO Rep. 7, 750–753. doi: 10.1038/sj.embor.7400769 16880817 PMC1525145

[B48] Seger M. Gebril S. Tabilona J. Peel A. Sengupta-Gopalan C. (2015). Impact of concurrent overexpression of cytosolic glutamine synthetase (GS1) and sucrose phosphate synthase (SPS) on growth and development in transgenic tobacco. Planta 241, 69–81. doi: 10.1007/s00425-014-2165-4 25213117

[B49] Szankowski I. Waidmann S. Degenhardt J. Patocchi A. Paris R. Silfverberg- Dilworth E.. (2009). Highly scab-resistant transgenic apple lines achieved by introgression of HcrVf2 controlled by different native promoter lengths. Tree Genet. Genom 5, 349–358. doi: 10.1007/s11295-008-0191-8

[B50] Telem R. S. Wani H. Singh N. B. Nandini R. Sadhukhan R. Bhattacharya S.. (2013). Cisgenics - a sustainable approach for crop improvement. Curr. Genomics 14 (7), 468–476. doi: 10.2174/13892029113146660013 24396278 PMC3867722

[B51] Thomsen H. C. Eriksson D. Moller I. S. Schjoerring J. K. (2014). Cytosolic glutamine synthetase: a target for improvement of crop nitrogen use efficiency? Trends Plant Sci. 19, 656–663. doi: 10.1016/j.tplants.2014.06.002 25017701

[B52] Townsend J. A. Wright D. A. Winfrey R. J. Fu F. Maeder M. L. (2009). High-frequency modification of plant genes using engineered zinc-finger nucleases. Nature 459 (7245), 442–445. 19404258 10.1038/nature07845 PMC2743854

[B53] Urriola J. Rathore K. S. (2015). Overexpression of a glutamine synthetase gene affects growth and development in sorghum. Transgenic Res. 24, 397–407. doi: 10.1007/s11248-014-9852-6 25417185

[B54] Vanblaere T. Flachowsky H. Gessler C. Broggini G. A. L. (2014). Molecular characterization of cisgenic lines of apple ‘gala’ carrying the Rvi6 scab resistance gene. Plant Biotechnol. 12, 2–9. doi: 10.1111/pbi.12110 23998808

[B55] Vanblaere T. Szankowski I. Schaart J. Schouten H. Flachowsky H. Broggini G. A. L.. (2011). The development of a cisgenic apple plant. J. Biotechnol. 154, 304–311. doi: 10.1016/j.jbiotec.2011.05.013 21663775

[B56] Van Gelder W. M. J. (1989). Steroidal glycoalkaloids in Solanum species: consequences for potato breeding and food safety (Netherlands: University of Wageningen).

[B57] Vignault C. Vachaud M. Cakir B. Glissant D. Dedaldechamp F. Buttner M.. (2005). VvHT1 encodes a monosaccharide transporter expressed in the conducting complex of the grape berry phloem. J. Exp. Bot. 56 (415), 1409–1418. doi: 10.1093/jxb/eri142 15809282

[B58] Walker A. R. Lee E. Bogs J. Mcdavid D. A. Thomas M. R. Robinson S. P. (2007). White grapes arose through the mutation of two similar and adjacent regulatory genes. Plant J. 49 (5), 772–785. doi: 10.1111/j.1365-313X.2006.02997.x 17316172

[B59] Weeks J. T. Ye J. Rommens C. M. (2008). Development of an in-planta method for transformation of alfalfa (*Medicago sativa*). Transgenic Res. 17 (4), 587–597. doi: 10.1007/s11248-007-9132-9 17851774

[B60] Williams L. E. Lemoine R. Sauer N. (2000). Sugar transporters in higher plants-a diversity of roles and complex regulation. Trends Plant Sci. 5 (7), 283–290. doi: 10.1016/s1360-1385(00)01681-2 10871900

[B61] Wormit A. Trentmann O. Feifer I. Lohr C. Tjaden J. Meyer S.. (2006). Molecular identifi cation and physiological characterization of a novel monosaccharide transporter from Arabidopsis involved in vacuolar sugar transport. Plant Cell. 18 (12), 3476–3490. doi: 10.1105/tpc.106.047290 17158605 PMC1785410

[B62] Zheng Y. Tian L. Liu H. Pan Q. Zhan J. Huang W. (2009). Sugars induce anthocyanin accumulation and flavanone 3-hydroxylase expression in grape berries. Plant Growth Regul. 58 (3), 251–260. doi: 10.1007/s10725-009-9373-0

[B63] Zuo J. Niu Q. W. Moller S. G. Chua N. H. (2001). Chemical-regulated, site specific DNA excision intransgenic plants. Nat.Biotechnol 19, 157–161. doi: 10.1038/84428 11175731

